# Rearing in strontium-enriched water induces vaterite otoliths in the Japanese rice fish, *Oryzias latipes*

**DOI:** 10.1098/rsos.230410

**Published:** 2023-06-14

**Authors:** Iki Murase, Tatsuhiko Kawamoto, Norikatsu Akizawa, Takahiro Irie

**Affiliations:** ^1^ Tropical Biosphere Research Center, University of the Ryukyus, Nishihara, Okinawa 903-0213, Japan; ^2^ Atmosphere and Ocean Research Institute, The University of Tokyo, Kashiwa, Chiba 277-8564, Japan; ^3^ Department of Geoscience, Faculty of Science, Shizuoka University, Shizuoka, Shizuoka 422-8529, Japan

**Keywords:** CaCO_3_ polymorphism, vaterite, aragonite, sagittal otoliths, strontium, crystalline otoliths

## Abstract

Sagittal otoliths, typically composed of aragonite, are frequently laid down rather as vaterite during growth in hatchery-reared fish populations. Sagittal vateritization is believed to impair individual hearing/balancing abilities, but the causal mechanism remains unclear. Here we experimentally demonstrated that rearing in Sr-rich water induces sagittal vateritization in the HdrR-II1 inbred strain of the Japanese rice fish, *Oryzias latipes*. Both sagittae were partly vateritized in 70% of individuals subjected to the Sr^2+^ treatment (*n* = 10), whereas fish reared in normal tap water showed no sagittal vateritization (*n* = 8). Our result is consistent with the theoretical prediction that vaterite becomes thermodynamically more stable than aragonite as the Sr^2+^ concentration in solution increases. A vateritic layer develops surrounding the original aragonitic sagitta in vateritized otoliths, some of which take on a comma-like shape. Electron probe microanalysis demonstrates that the vateritized phase is characterized by lower Sr^2+^ and higher Mg^2+^ concentrations than the aragonitic phase. It is unlikely that increased environmental Sr^2+^ is responsible for the sagittal vateritization in farmed fish. However, our findings likely help to establish an *in vivo* assay using *O. latipes* to understand the physiological process underlying the sagittal vateritization in farmed fish.

## 1. Introduction

Teleost fishes have three pairs of otoliths in their inner ear, termed sagittae, lapilli and asterisci, which are composed mainly of calcium carbonate (CaCO_3_) [1]. CaCO_3_ has three anhydrous crystalline polymorphs under ambient conditions: calcite, aragonite and vaterite. Sagittae and lapilli of teleost species are commonly made of aragonite, whereas asterisci are of vaterite (reviewed by [2]). Nevertheless, some individuals have abnormal sagittae in which aragonite is partly replaced with vaterite [3–5]. This phenomenon has been reported in several salmonid species (e.g. *Oncorhynchus kisutch*; [6]), Atlantic herring (*Clupea harengus*; [5]), European eel (*Anguilla anguilla*; [7]) and lake sturgeon (*Acipenser fulvescens*; [8–10]). Looking at patterns within a species, individuals with vateritic sagittae are seen in wild populations, but the proportions are much greater in farmed fish [6,11]. The physiological mechanism controlling polymorphism in otolith CaCO_3_ is poorly understood, except that a small number of relevant proteins have been reported (reviewed in Discussion). Yet the ionic composition of and organic molecules in the endolymphatic fluid surrounding otoliths seem to be critically responsible for the thermodynamics that determines which polymorph is precipitated [12–15].

In species that normally have aragonitic sagittae, vateritization of sagittae is thought to negatively affect individual growth and/or survival [16,17]. In hatchery-reared Atlantic salmon (*Salmo salar*), vateritic sagittae lead to lower return rates from ocean migration, when compared to recaptured adults with normal aragonitic otoliths [18]. This could be direct evidence that otolith vateritization reduces the oceanic survival potential of anadromous fish. There is also experimental evidence that fish with vateritized sagittae exhibit escape behaviour quantitatively distinguishable from the individuals with normal otoliths, which may be detrimental to predator avoidance in the wild [17]. As vaterite is less dense than aragonite, otolith mass asymmetry may cause a loss of auditory function when one of two sagittae is vateritized [16]. All of these studies suspect that stressful hatchery conditions are responsible for the sagittal vateritization, simply because the proportion of hatchery-reared individuals with vateritic sagittae is higher than their wild born conspecifics [18]. This means that there is a strong social demand for establishing a fish farming technique that prevents sagittal vateritization.

**Figure 1.**
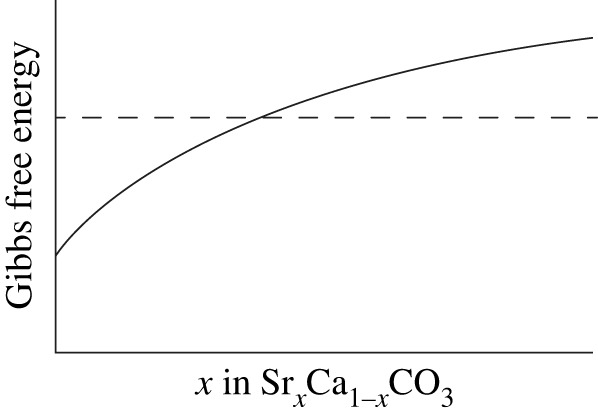
A hypothetical model behind our working hypothesis, evaluated as the thermodynamic stability of calcium carbonate polymorphs as a function of Sr^2+^ concentration in the solution. The horizontal axis represents the relative abundance of Sr in aragonite as Sr*_x_*Ca_1−*x*_CO_3_. A lower value of the Gibbs free energy corresponds to increased thermodynamic stability of the focal polymorph. Vaterite (dashed line) becomes thermodynamically more stable than aragonite (solid line) when Sr concentration exceeds a threshold value. The positive relationship between the Sr concentration in aragonite and the Gibbs free energy is reported by [27] and [28]. A dashed line is horizontal because no information is available on how the free energy depends on the Sr concentration in vaterite.

Of the three crystalline polymorphs of CaCO_3_, calcite is thermodynamically the most stable phase at room temperature and pressure, meaning that calcite stabilizes when CaCO_3_ precipitates from supersaturated solutions (e.g. [19]). However, this principle appears to contradict the fact that aragonitic biominerals are commonly precipitated by marine calcifiers [20]. It is now widely accepted that aragonite rather than calcite precipitates when the solution (e.g. seawater) contains a certain concentration of Mg^2+^, which has been repeatedly demonstrated experimentally (e.g. [20–22]). The theory for a quantitative understanding of this phenomenon is still far from complete (e.g. [23,24]), but a qualitative explanation has been given based on the kinetic property that calcite readily forms a solid solution with Mg^2+^ that substitutes for Ca^2+^ in the hexagonal lattice whereas aragonite does not (e.g. [25,26]). By analogy, we hypothesized that the relationship between calcite and aragonite in the presence of Mg^2+^ could be applied to the relationship between aragonite and vaterite in the presence of Sr^2+^ (figure 1). This is because Sr^2+^ can be better accommodated in the orthorhombic crystal structure of aragonite (e.g. [25,26,29]), but cannot in the hexagonal structure of vaterite [30]. Our proposal is compatible with the result of numerical simulations by [27], showing that aragonite becomes thermodynamically less stable as the concentration of Sr^2+^ increases, to the extent that Sr^2+^ is lower than Ca^2+^ in the solution. More recently, the prediction by [27] has been supported experimentally by [28]. These findings gave us the idea that sagittal vateritization may more likely occur as the endolymph surrounding sagittae contains a higher amount of Sr^2+^.

The purpose of this study is to establish a rearing technique to induce vateritic sagittae in the fish species normally forming aragonitic sagittae. Such a technique provides a methodological framework making it possible to quantitatively evaluate the impact of hatchery-reared fish with vateritic sagittae on their populations and ecosystems, as well as to investigate the effect on their individual fitness. Based on the thermodynamic idea proposed above, we formulated a working hypothesis that rearing fish in Sr-rich freshwater causes vateritization of sagittae, because previous studies have suggested that divalent cations incorporated into otoliths are mainly derived from ambient water rather than food [31–33]. The hypothesis was tested experimentally by rearing the Japanese rice fish (*Oryzias latipes*), which has long been used as a model organism for genomics in biology. Especially, we selected the inbred HdrR-II1 strain aiming to minimize the impact of intraspecific genetic variation in the vateritizing response. As a result of the experiment, our working hypothesis was accepted, meaning that a rearing technique to induce partly vateritic sagittae was successfully established. We believe that our procedure promotes the understanding of the atomic/molecular basis behind sagittal vateritization by means of applying various omics approaches developed in molecular biology to the inbred strain of *O. latipes*.

## 2. Material and methods

### 2.1. Rearing setup

Experimental individuals of the Japanese rice fish, *Oryzias latipes*, were produced by random mating among 3 male and 3 female parental individuals from the HdrR-II1 inbred strain (strain ID: IB178) provided by the National BioResource Project at the National Institute of Genetics (Mishima, Japan). Fertilized eggs were individually kept in gently aerated, cylindrical plastic bottles (1100 ml, 96 mm in diameter) placed in a temperature-controlled water bath under natural light conditions.

The rearing bottles were randomly assigned into two groups: Sr-rich treatment and control (rearing with normal tap water). Rearing water in the Sr-rich treatment was prepared by adding 24.0 mg of strontium chloride hexahydrate (SrCl_2_·6H_2_O) to 1.0 l of tap water. We determined this dosage so as to simply tune to the Sr^2+^ concentration in seawater, because no prior information was available for the minimum Sr^2+^ concentration to induce vateritic otoliths in *O. latipes*. Analyses using an inductively coupled plasma atomic emission spectroscopy indicated that the rearing tap water contained 20.7 mg l^−1^, 4.5 mg l^−1^ and 75.3 μg l^−1^ of Ca^2+^, Mg^2+^ and Sr^2+^, respectively (*n* = 6; ICP-AES, SPECTRO BLUE FMX26, Spectro, for Ca^2+^ and Mg^2+^; ICP-MS Agilent 7700x, Agilent Technologies, for Sr^2+^). Accordingly, the water Sr/Ca value of the Sr-rich treatment was 176.2 mmol mol^−1^, which was on average 106 times greater than that of the control water (=1.67 mmol mol^−1^). For the same species, the Sr/Ca of ambient water evaluated as the median lethal concentration (LC_50_) was ca. 13 mol mol^−1^ [34], meaning that the water Sr/Ca in our Sr-rich treatment was less than 1.4% of the reported LC_50_ value. Looking at the toxicity of Sr as a metal ion, the concentration in our Sr-rich treatment is less than 2% of the LC_50_ (=415 mg l^−1^) experimentally estimated in this species [34].

Average rearing temperature (± s.d.) was 23.8 ± 0.05°C according to the hourly data monitored by a HOBO Pendant Temperature Data Logger UA-001-64 (Onset Computer Corporation, Bourne, MA USA). Each fish was fed a powdered diet (DCM Medaka Food, Keiyo Co., Ltd., Japan) during the first 10 days after hatching, and then first instar *Artemia* larvae (hatched in natural seawater at 28°C) twice a day. To maintain the water quality, 20% of water in each rearing bottle was replaced with newly prepared water once a week.

The numbers of fertilized eggs assigned to the Sr-rich treatment and the control were 18 and 11, but only 10 and 8 individuals were alive 3 weeks after hatching, respectively (i.e. the initial mortality rate of the Sr-rich treatment was approximately 1.6 times greater than that of the control). Exceptionally, one individual in the Sr-rich treatment died at the age of 114 days after hatching from an unknown cause (table 1). The other 17 fish were killed by being submerged into iced water (0–4°C) at 121–146 days of age.

**Table 1.** Hatching date, age at the end of rearing (days), final standard length (mm), final body weight (g), and CaCO_3_ polymorphs of left (L) and right (R) otoliths (weight in mg). A and V represent the purely aragonitic otolith and partly vateritized otolith, respectively. The last column contains the critical Sr/Ca value at a transition from aragonite to vaterite in each otolith.

ID	hatching date	age (days)	SL (mm)	BW (g)	otolith (mg)	Sr/Ca (mmol mol^−1^)
(a) control						
C01	21/11/2020	142	17.78	0.055	L: A (0.079)	
					R: A (0.083)	
C02	22/11/2020	141	18.34	0.092	L: A (0.096)	
					R: A (0.096)	
C03	25/11/2020	138	16.26	0.071	L: A (0.088)	
					R: A (0.093)	
C04	17/11/2020	146	17.50	0.079	L: A (0.078)	
					R: A (0.088)	
C05	4/12/2020	129	17.39	0.092	L: A (0.097)	
					R: A (0.087)	
C06	12/12/2020	121	16.88	0.066	L: A (0.076)	
					R: A (0.064)	
C07	12/12/2020	121	17.09	0.072	L: A (0.081)	
					R: A (0.073)	
C08	12/12/2020	121	16.89	0.070	L: A (0.076)	
					R: A (0.087)	
(b) Sr-rich						
S01	2/12/2020	114	10.88	0.018	L: V (0.052)	127.8
					R: V (0.051)	148.7
S02	22/11/2020	142	17.08	0.074	L: V (0.074)	96.3
					R: V (0.085)	112.2
S03	20/11/2020	144	17.08	0.072	L: V (0.091)	110.6
					R: V (0.082)	116.9
S04	24/11/2020	140	16.74	0.083	L: V (0.124)	130.7
					R: A (0.106)	
S05	24/11/2020	140	17.73	0.095	L: V (0.105)	94.4
					R: V (0.085)	109.3
S06	2/12/2020	131	16.73	0.065	L: V (0.078)	103.5
					R: V (0.068)	125.2
S07	24/11/2020	140	16.80	0.070	L: V (0.077)	127.2
					R: V (0.092)	132.6
S08	2/12/2020	131	16.40	0.072	L: V (0.103)	130.0
					R: A (0.087)	
S09	2/12/2020	129	16.19	0.065	L: V (0.090)	127.1
					R: A (0.077)	
S10	5/12/2020	129	17.43	0.078	L: V (0.109)	100.7
					R: V (0.097)	118.4

### 2.2. Extraction and processing of otoliths

**Figure 2.**
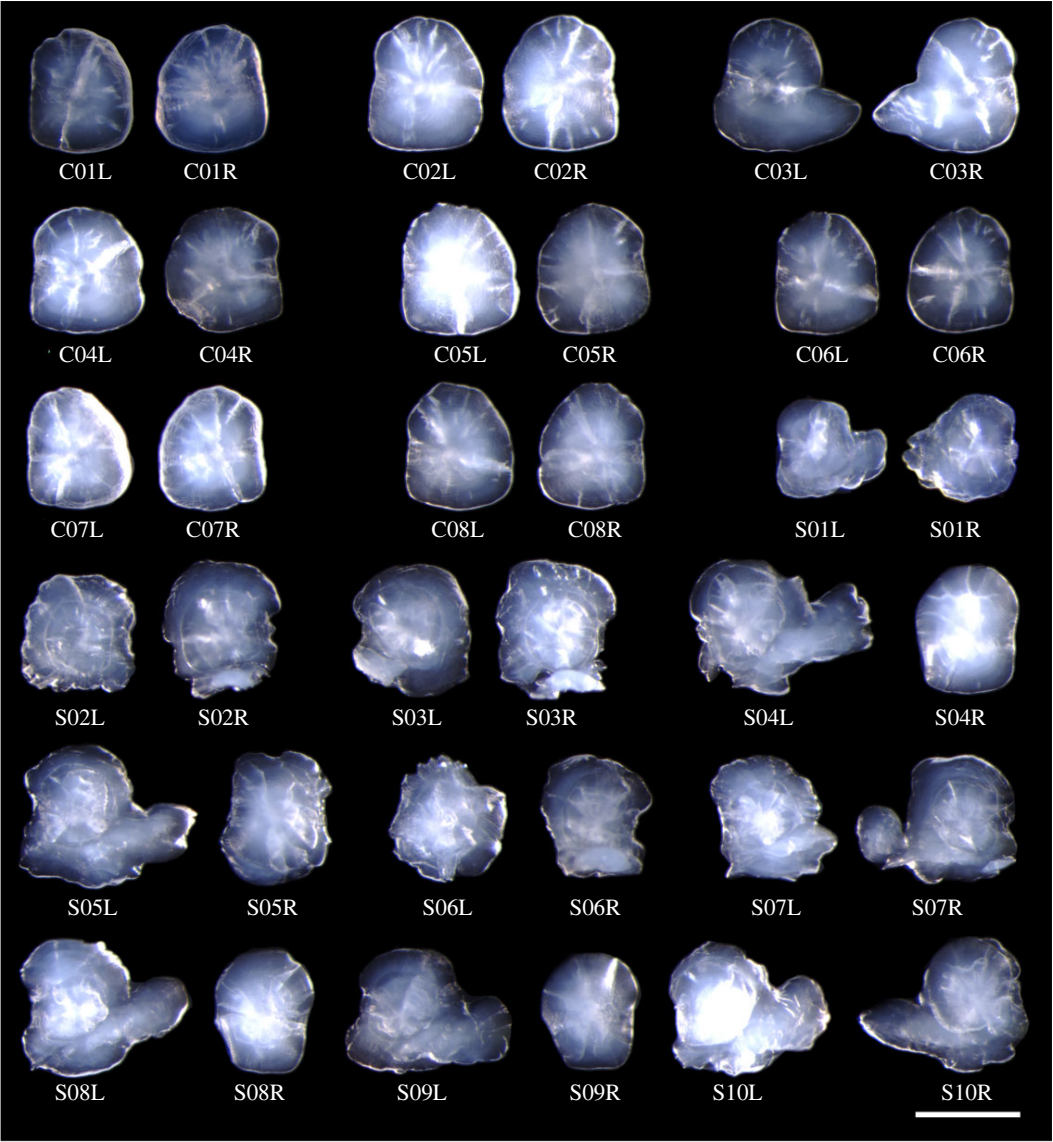
Left-sided (L) and right-sided (R) sagittal otoliths of experimental individuals. C and S represent the control and Sr-rich treatment groups, respectively. Scale bar indicates 500 µm.

Standard body length (SL) and wet body weight (BW) of each individual were measured using digital calipers (mm) and an electronic scale (g), respectively, for calculating the mean somatic growth rate for the experimental period. Sagittal otoliths were physically extracted from both sides of the body (figure 2), and individually weighed using an electronic scale (mg, BM-20, A&D Company, Limited). The bottom surface of a cone-shaped sagitta was attached onto a glass slide with double-sided tape, and then embedded in epoxy resin (EpoFix; Struers). After hardening, the top surface of epoxy resin was ground to a flat surface with a circular diamond saw blade (Isomet 5000, Buehler) and a diamond cup-wheel (Discoplan-TS; Struers). The flattened surface was mounted on another glass slide using epoxy glue (Quick 30, Konishi, Osaka, Japan). The bottom surface of a cone-shaped sagitta was ground with SiC foil (grit #1200, #2000, #4000; Struers) to expose the core, and then buffed with an active oxide polishing suspension (OP-S suspension; Struers) on a polishing wheel fitted with a semi-automatic specimen mover (MD-Chem and RotoPol-35/PdM-Force-20; Struers). The glass slide with the otolith was cleaned in an ultrasonic bath, rinsed for 1 min with 99% ethanol, for 1 min with deionized water, and then dried by placing in a vacuum desiccator (at 0.1 atm) at room temperature for 24 h.

### 2.3. Raman spectroscopic analysis

The polymorphic forms of otolith calcium carbonate were identified using a confocal Raman microscope (WITec alpha300R, Ulm, Germany) at the Department of Geoscience, Faculty of Science, Shizuoka University, Japan. The excitation laser beam was obtained through 20× or 100× objective lens depending on the target polymorphic form, with a 488 nm laser at 2 mW on sample position using 100× objective lens, 600 g mm^−1^ gratings and CCD detector from 104 to 3652 cm^−1^ spectral range. Calibration in Raman shift units (expressed in cm^−1^) was performed with naphthalene at 1382.2, 1022, 764 and 3056 cm^−1^ peaks. In agreement with [35] and [36], vaterite was characterized by a relatively broad peak consisting of triple bands at 1074, 1081 and 1090 cm^−1^, which made it possible to distinguish vaterite from aragonite, indicating a sharp peak at 1085 cm^−1^ (electronic supplementary material, figure A1). Bimodal peaks around 707 cm^−1^ unique to aragonite and around 295 cm^−1^ unique to vaterite were also useful for discrimination (electronic supplementary material, figure A1). Only two (C08L and S09L) out of 36 sagittae were subjected to Raman mapping based on Raman spectra (electronic supplementary material, figure A1). No quantitative data are collected from the other sagittae, because polymorph identification for the entire sample (*n* = 36) was based on divalent cation concentrations (see below).

### 2.4. Electron probe microanalyser analysis

Concentrations of some divalent cation species were sclerochronologically quantified from the core to the edge of each otolith, because the vateritic phase is characterized by both lower strontium (Sr) and higher magnesium (Mg) concentrations than the aragonitic phase [5]. Prior to the analysis, the upper surface of the specimens was coated with platinum-palladium for 60 s in an ion-beam sputter coater (Hitachi E-1030) to enhance electrical conductivity. Chemical composition of the otoliths was obtained using a JEOL JXA-8230 electron probe microanalyser (EPMA) at the Atmosphere and Ocean Research Institute, University of Tokyo (see [37]). The wavelength-dispersive spectrometry (WDS) method was used to measure Ca, Mg and Sr concentrations with the electron beam of 5 µm in diameter with 5 µm intervals. Exposure times were set at 10 s for peaks and 5 s for background measurements with an accelerating voltage of 15 kV and a beam current of 12 nA. Wollastonite (CaSiO_3_), magnesium oxide (MgO), and strontium titanate (SrTiO_3_) were used as standard materials for the ZAF correction. Otoliths collected from all 18 individuals (*n* = 36) were subjected to the EPMA analysis.

### 2.5. Statistical analysis

Dependencies of sagittal weight, standard body length and wet body weight on experimental conditions (designated as fixed-effect factors) and covariates were statistically analysed by fitting ANCOVA models. In all analyses, interaction terms were statistically nonsignificant (all *p* > 0.05) and were removed from the final models. In the ANCOVA of sagittal weight, the number of individuals instead of the number of otoliths was used to calculate the second degrees of freedom in *F*-tests to avoid pseudoreplication, because otoliths collected from the same individuals are unlikely to be statistically independent in terms of residual weight. All statistical analyses were conducted in the JMP Pro statistical package (v. 13 for Windows; SAS Institute) and Mathematica (v. 11.0.1 for Windows; Wolfram Research).

## 3. Results

**Figure 3.**
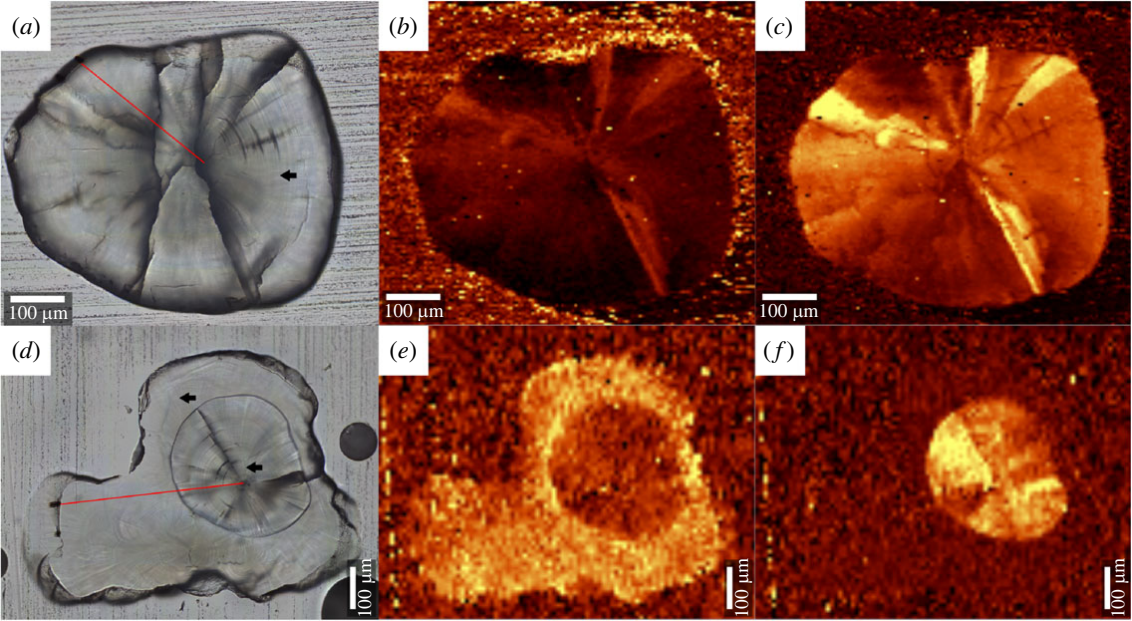
Raman mapping images of sagittal otoliths from the individuals assigned to the control (*a,b,c*) and Sr-rich treatment (*d,e,f*) groups (corresponding to specimens C08L and S09L, respectively). Panel (*a*) is a transmitted light photograph of the sagitta from C08L, in which the arrow indicates the position of the Raman spectroscopic measurement (see electronic supplementary material, figure A1(A)). The counterpart to S09L is shown in panel (*d*); upper and lower arrows represent the positions where Raman spectroscopic measurements were conducted (see electronic supplementary material, figure A1(B)), corresponding to the vateritic and aragonitic phases, respectively. Red straight lines indicate the positions subjected to the EPMA analysis (figure 5). (*b*) and (*e*) are based on the Raman shift signal intensity at 295 ± 35 (cm^−1^), which is a specific peak shift characteristic to vaterite. (*c*) and (*f*) highlight the Raman shift signal intensity at 707 ± 10 (cm^−1^), which is a peak shift characteristic to aragonite.

The Raman spectrum for the aragonitic phase of a sagitta collected from an individual in the control group (C08L) is shown in electronic supplementary material, figure A1(A), which corresponds to the measurement at the spot indicated by the arrow in figure 3*a*. Electronic supplementary material, figure A1(B) illustrates spectra for both aragonitic and vateritic phases, measured from an individual subjected to the Sr-rich treatment (S09L), as indicated in figure 3*d*. Individuals with partly vateritized sagittae occurred only in the Sr-rich treatment (table 1) in contrast to the finding that all individuals consistently had aragonitic otoliths when reared in the normal tap water (table 1). Under the Sr-rich condition, 7 individuals had partly vateritized sagittae on both sides of the body and the other 3 individuals had a partly vateritized sagitta only on the left-hand side (table 1). A Fisher's exact test rejected the null hypothesis that Sr-rich treatment has no impact on the frequency of sagittal vateritization (*p* < 0.0001).

Vateritic sagittae are typically characterized by a comma-like shape (figure 2; e.g. S07R), in which an aragonitic portion precipitated for a certain period of time after birth and the periphery is coated by a vateritic layer in a concentric manner (figure 3). Concentric growth rings are found also in the thickened part of the vateritic layer, suggesting the possibility that the partly vateritized sagitta is fused with an asteriscus (see Discussion for further evidence). This pattern is found in 14 out of the 17 sagittae with a vateritized layer (electronic supplementary material, figure A2).

**Figure 4.**
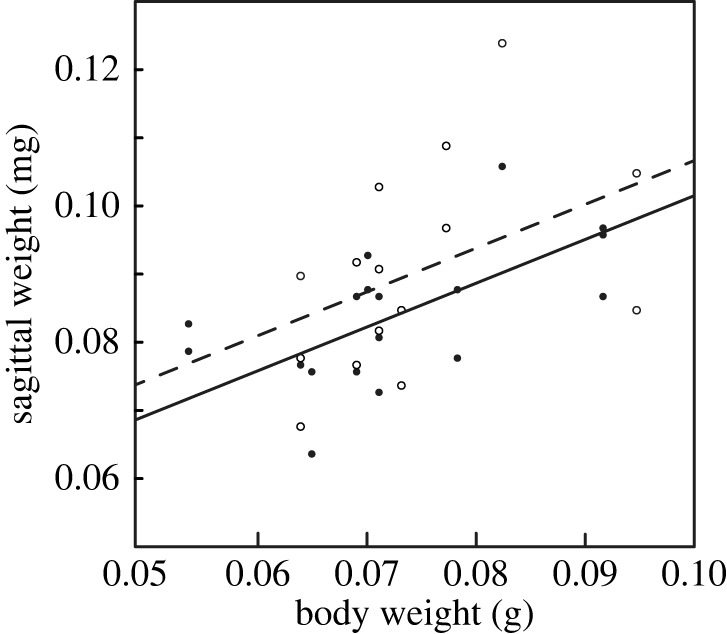
Relationships between wet body weight (g) and sagittal weight (mg) compared between aragonite (closed circles with a solid line) and partly vateritized otoliths (open circles with a dashed line). Regression lines are derived from the ANCOVA model without the interaction effect assumed. Data points from S01 (BW = 0.018 g) are out of the frame (table 1).

Sagittal weight was fitted by an ANCOVA model with the polymorphic type of otolith as a fixed-effect factor (i.e. A/V in table 1) and body weight as a covariate. This model was capable of better describing the increase in otolith weight along the ontogeny (*R*^2^ = 0.53) compared to the models with body length (*R*^2^ = 0.36) being assigned to the covariate instead of body weight. When adjusting for the effect of body weight, partly vateritized sagittae were approximately 6% heavier than aragonitic ones (d.f. = 1, 15; *p* > 0.05; *n* = 36; figure 4). However, this does not mean that partly vateritized sagittae grew larger, because some otoliths were subjected to the weight gain by fusing with an asteriscus.

**Figure 5.**
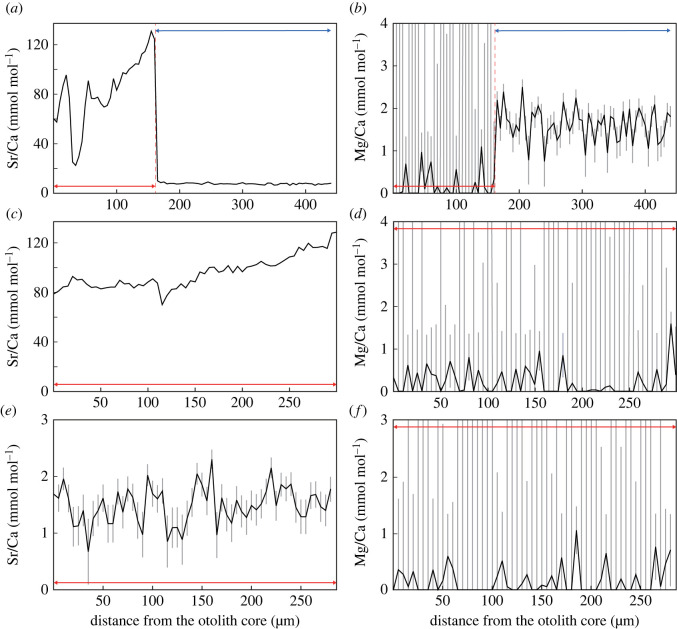
Sr/Ca and Mg/Ca values measured along straight lines from sagittal core to edge (see electronic supplementary material, figure A5). (*a*) Sr/Ca and (*b*) Mg/Ca profiles of an individual reared in Sr-rich water (S09L). Aragonite-to-vaterite transition was observed (the boundary is highlighted with a red line). (*c*) Sr/Ca and (*d*) Mg/Ca profiles of an individual reared in Sr-rich water (S04R). An aragonitic phase was maintained throughout the experimental period. (*e*) Sr/Ca and (*f*) Mg/Ca profiles of an individual reared in the normal tap water (C08L). No vateritization occurred. Vertical bars (grey) represent measurement errors evaluated as standard deviation (±s.d.). Red and blue horizontal lines indicate the zones identified as the aragonitic and vateritic phases by the EPMA approach, respectively (figure 3). Notice that the transect reflects the elemental ratio change along the ontogeny in the aragonitic phase, but may not in the vateritic phase because the transect passes through the cross-sectional surface of an asteriscus fused to the sagitta in some vateritized otoliths. This is important when assuming that the asteriscus is concentrically precipitated prior to the fusion with a vateritized sagitta.

Individuals exposed to the Sr-rich treatment are characterized by the otolith elementary profile that Sr/Ca gradually increases along the ontogeny throughout the aragonitic phase (figure 5*a,c*). The range and slope of the increase vary among individuals; for example, S07L shows an increase from 83 to 134 mmol mol^−1^, whereas the change in S05L is less steep (from 90 to 97 mmol mol^−1^) (electronic supplementary material, figure A3). The critical Sr/Ca value at an aragonite-to-vaterite transition was also variable among individuals, ranging from 94 to 149 mmol mol^−1^ (table 1). After the transition to the vateritic phase, Sr/Ca continued to drift around 6 to 10 mmol mol^−1^ (figure 5*a*) and a detectable level of Mg incorporation was observed (approx. 3.0 mmol mol^−1^, figure 5*b*, electronic supplementary material, figures A3 and A4). The boundary between the two phases is clear in the sense that the mixed phase of aragonite and vaterite are not found. These patterns are not seen in the individuals reared in the normal tap water, in which otolith Sr/Ca is within the range between 0.5 and 3.0 mmol mol^−1^ (figure 5*e*, electronic supplementary material, figure A3 and A4). One individual in the control group (C03) formed sagittae similar in shape to those fused with asterisci (figure 2), but the EPMA analysis demonstrated that the protruding part in C03 otoliths was purely aragonitic (i.e. fusion with an asteriscus was unlikely). In both groups of fish, sagittal Mg/Ca in the aragonitic phase remained below 1 mmol mol^−1^ (figure 5*b,d,f*; electronic supplementary material, figure A4).

In three individuals assigned to the Sr-rich treatment, sagittae on the right-hand side remained aragonitic throughout the experimental period (table 1). Sr/Ca values continued to increase throughout the rearing period in these sagittae (figure 5*c*; see also S04R, S08R and S09R in electronic supplementary material, figure A3).

On the age-adjusted mean SL at the end of rearing, individuals assigned to the Sr-rich treatment were 2.7% smaller than the adjusted mean SL of those to the control group. However, this difference was not significantly different (*p* > 0.05; *n* = 17 excluding one outlier) in an ANCOVA with the treatment as a fixed-effect factor and age at collection as a covariate. The difference in age-adjusted mean BW at collection was negligible between the control and Sr-rich groups (resulting in a decrease of 0.6%), which was statistically supported by applying an ANCOVA model on the cubic root of BW (*p* > 0.05; *n* = 17 excluding one outlier).

## 4. Discussion

Our experiment successfully demonstrated that rearing with Sr-rich water induces sagittal vateritization in *Oryzias latipes*. Notably, our result is qualitatively compatible with the prediction based on the thermodynamic stability model predicting that vaterite preferably precipitates over aragonite at the presence of high concentrations of Sr^2+^ in the solution (figure 1). This does not necessarily mean that the thermodynamic model is experimentally supported, because the aragonite-to-vaterite transition in sagittae is also likely to be caused by some physiological stress (e.g. [6,38]). High levels of Sr^2+^ might operate as a stressor responsible for the sagittal vateritization in our experiment. These two possibilities are not mutually exclusive, but the empirical adequacy of the Sr^2+^-based thermodynamic model should be carefully checked in the future study. This could be achieved if a divalent cation that readily forms a solid solution in vaterite (but not in aragonite) is used in the rearing experiment in place of Sr^2+^. For example, Mn^2+^ might be an excellent candidate, because it is also toxic to *O. latipes* [34]. Mn^2+^ is thermodynamically not expected to cause the aragonite-to-vaterite transition, considering that its ionic radius (81 pm for VIII coordinated [30]) is much smaller than that of Ca^2+^ (112 pm for VIII coordinated) and Sr^2+^ (132 pm for VIII coordinated [30]).

Albeit the sceptical argument above, we propose a piece of evidence that may support the possibility that a high Sr^2+^ concentration in endolymph is responsible for sagittal vateritization. In freshwater fishes, both Ca^2+^ and Sr^2+^ actively pumped into the blood plasma from ambient water via epithelial transporters are incorporated into the endolymph surrounding otoliths; however, their affinity to Sr^2+^ is much lower than that to Ca^2+^ [39,40]. Our EPMA data on the aragonitic phase of vateritized sagittae strongly suggest that elevated endolymphatic Sr/Ca is involved in the vateritization (figure 5*a*, electronic supplementary material, figure A3). The sagittal Sr/Ca at the aragonite-to-vaterite transition is variable among individuals as well as between both sagittae of the same individual (table 1), which rejects the possibility that the transition occurs consistently when a sagittal Sr/Ca reaches a threshold with a fixed value. The aragonite-to-vaterite transition may be a stochastic event and its likelihood may increase with increasing endolymphatic Sr/Ca. Otherwise, the observed variability in the sagittal Sr/Ca level may simply reflect the variation in Sr^2+^-affinity of transmembrane Ca^2+^-transporters among individuals [41,42] as well as between the two saccules of the same individual.

**Figure 6.**
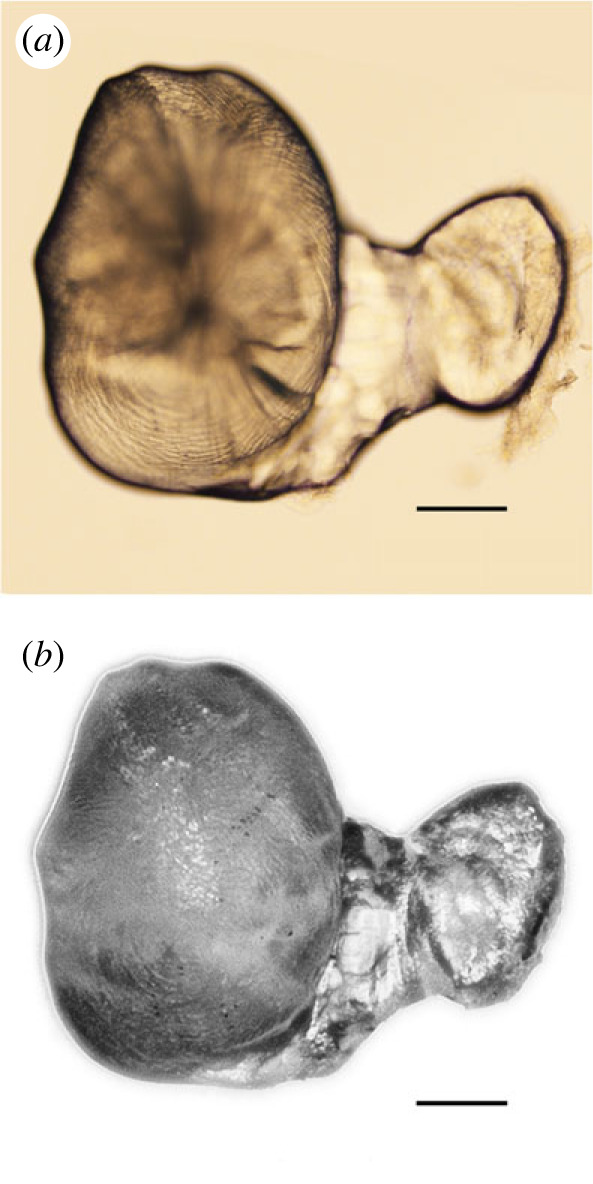
A partly vateritized sagitta (left) fused with an asteriscus (right) extracted from an individual reared in Sr-rich water (a specimen from a preliminary experiment using a wild type *Oryzias latipes*). Photographed with (*a*) the transmitted light and (*b*) the reflected light. Bars represent 100 μm.

Another important finding in this study is that half of partly vateritized sagittae (9 out of 17) showed a comma-like shape (figure 2). Microscopic observations of growth lines running in the vateritic layer suggest that the malformed otoliths may result from the fusion between a vateritized sagitta and an asteriscus (electronic supplementary material, figure A2). The same phenomenon occurs in wild-type individuals of *O. latipes* (figure 6). In this species, asterisci are composed of vaterite and concentric growth rings are observed (electronic supplementary material, figure A6). A similar concentric pattern was found within the vateritic layer in 14 out of 17 vateritized sagitta, supporting the possibility of the fusion with an asteriscus. This phenomenon seems to be closely related to the previous finding that an asteriscus shrinks and then disappears when the neighbouring sagitta is vateritized [43]. It is an open question how frequently the fusion actually occurs in wild *Oryzias latipes*, because our findings are based on the results from offspring produced by breeding among a small number of inbred-strain parents (*n* = 6). Surveys in wild and hatchery-reared populations are needed to gain a quantitatively unbiased understanding of how detrimental the fusion between sagittae and asterisci is to individual fitness and population growth in the field.

Sagittal vateritization occurs with notoriously high frequency in hatchery salmonids, but the causal mechanism has not been identified. Contrary to the result of our experiment, we do not consider that a high Sr^2+^ concentration in ambient water is responsible for this phenomenon. It is possible that the Sr/Ca values in the groundwater used at hatcheries exceed the critical Sr/Ca values at which the aragonite-to-vaterite transitions occurred in our experiment (≈ 0.1 mol mol^−1^) if bedrocks are Sr-rich in aquifers [44]. However, such high Sr/Ca values have not been reported from the aragonitic phase of otolith profiles in hatchery-reared salmonids (Sr/Ca < 2 mmol mol^−1^ [30,45,46]). High levels of phosphate, rather than Sr^2+^, in ambient water may be responsible for the vateritization (see [47]) because phosphate is a calcium chelating agent and thus affects the thermodynamic landscape for CaCO_3_ polymorphism [48]. Sagittal vateritization has also been suspected to be caused by rapid somatic growth [38,49], higher metabolic rates [6], increased thyroid hormone levels [50], longer photoperiod [38] and a gradient of chemical composition [51] or a higher [CO_3_^2−^]/[Ca^2+^] value [52] in endolymph as well as genetic components [53,54]. In the following paragraphs, we will propose some viewpoints that might be necessary for a proper understanding of the sagittal vateritization occurring in fish populations.

It is unquestionable that the CaCO_3_ precipitation in otoliths is under strong biological control [14,15], in the sense that actively synthesized organic molecules (i.e. proteins and other organic constituents) profoundly affect the thermodynamic landscape among the polymorphs by regulating crystal deposition. Accumulating evidence suggests that the nucleation and polymorph control of otoliths are substantially mediated by intrinsically disordered proteins such as OMM-64 [55] and Starmaker [53,56,57]. We are still far from a complete understanding of how specific proteins control the aragonite-vaterite polymorphism of otoliths, but an *in vitro* experiment demonstrated that the absence of a short-chain collagen, otolin-1, leads to the precipitation of vaterite at the presence of OMM-64 [13]. It is very likely that an intercellular heterogeneity in gene expression patterns explains the polymorphic difference between sagittae and asterisci [15]. Environmentally induced expression/suppression of such genes might be responsible for the sagittal vateritization observed in this study.

These arguments invoke an idea that vateritic otoliths may have some adaptive significance in wild populations. In other words, we surmise that the formation of aragonitic otoliths might be disadvantageous under a particular circumstance, although no direct evidence has been presented to support this idea. From the viewpoint of evolutionary ecology, sagittal vateritization could be regarded as a phenotypic plasticity that allows the rapid construction of less calcareous, protein-rich, sparse otoliths in exchange for a greater energetic cost, instead of slowly precipitating small-sized aragonitic otoliths with a higher density. This might be adaptive particularly under Ca-depleted conditions. There are multiple pieces of circumstantial evidence indirectly supporting our view.

In a comparison of pure CaCO_3_ crystals, vaterite is 10.9% and 2.3% lighter in density than aragonite and calcite, respectively [58], meaning that a specific volume of crystals is built up by a smaller number of Ca^2+^. When comparing otoliths with the same volume, vateritic otoliths contain a larger amount of protein than aragonitic otoliths [59]. Therefore, a vateritic otolith may require more energy to construct than an aragonitic otolith does because the synthesis of the organic matrix is energetically more costly than CaCO_3_ precipitation [60,61] as well as because vaterite possesses a higher free energy than aragonite (e.g. [62]). In this context, the sagittal vateritization observed in our experiment is better understood as a plastic response to the ‘relative’ Ca^2+^ deficiency in endolymph, caused by the increased concentration of Sr^2+^ (i.e. decreased [Ca^2+^] relative to [total divalent cations] in endolymph could be a cue of vateritization).

There is also evidence from experimental ecology against the hypothesis that sagittal vateritization unconditionally lowers the individual fitness. Experimental studies using juvenile Atlantic salmon have clearly demonstrated that faster somatic growth is positively associated with the occurrence of vateritic sagittae [38,49], suggesting that vateritization occurs when somatic growth unacceptably outpaces the maximum growth of aragonitic sagittae. In field experiments with hatchery-reared fry released in a river, the sagittal vaterite:aragonite ratio for the individuals found in stomach samples of predators (brown trout) was consistently smaller than the ratio at the time of release [49]. The ratio increased in the recaptured individuals that survived in the wild river for 2–3 months [49]. These results strongly suggest that sagittal vateritization does not decrease the gross survival rate in wild populations, although body size rather than sagittal CaCO_3_ polymorphism is presumably the principal determinant of predation susceptibility [49].

In addition, the intraspecific variation that a small proportion of individuals have vateritic sagittae has been repeatedly reported from wild panmictic populations of non-farmed species (e.g. blackbellied angler *Lophius budegassa*, [63]; *Pagellus acarne* and three other marine species, [64]). No one has been able to provide a convincing explanation for the question of why alleles responsible for the sagittal vateritization are not selected out by natural selection, if the development of vateritic sagittae unconditionally leads to reduced fitness.

In summary, the present study demonstrated that sagittal vateritization can be artificially induced by controlling the chemical composition of rearing water. Our results suggest that an experimental approach using chemically adjusted water is helpful to specify the environmental factors behind the sagittal vateritization occurring in freshwater hatchery-reared fish, as well as to correctly evaluate the impact of vateritic sagittae on the individual performance. Sagittal vateritization has been much less commonly reported from marine aquaculture fishes (but see [5,47,59]) compared to freshwater ones, which raises an untested hypothesis that marine fishes rarely vateritize sagittae because Ca^2+^ is inexhaustibly supplied from seawater. Another remark is that *O*. *latipes* provides an excellent model system for the application of rapidly advancing techniques in genomics and proteomics, which seems capable of bridging the gap between the classical thermodynamic stability model of CaCO_3_ polymorphism and the accumulating evidence that organic molecules modify the energy landscape by regulating biomineralization processes. Future studies are expected to investigate the possibility that forming vateritic rather than aragonitic sagittae is advantageous under particular environmental conditions, for example, leading to a limited Ca supply to the endolymph surrounding otoliths.

## Data Availability

The data are available from the Dryad Digital Repository: https://doi.org/10.5061/dryad.gmsbcc2rj [65]. Supplementary material is available online [66].
